# Effect of crystallinity and related surface properties on gene expression of primary fibroblasts

**DOI:** 10.1039/d1ra07237d

**Published:** 2022-02-01

**Authors:** Dorota Kołbuk, Marzena Ciechomska, Oliwia Jeznach, Paweł Sajkiewicz

**Affiliations:** Institute of Fundamental Technological Research, Polish Academy of Sciences Pawińskiego 5b 02-106 Warsaw Poland dkolbuk@ippt.pan.pl; National Institute of Geriatrics, Rheumatology and Rehabilitation Spartańska 1 02-637 Warsaw Poland

## Abstract

The biomaterial-cells interface is one of the most fundamental issues in tissue regeneration. Despite many years of scientific work, there is no clear answer to what determines the desired adhesion of cells and the synthesis of ECM proteins. Crystallinity is a characteristic of the structure that influences the surface and bulk properties of semicrystalline polymers used in medicine. The crystallinity of polycaprolactone (PCL) was varied by changing the molecular weight of the polymer and the annealing procedure. Measurements of surface free energy showed differences related to substrate crystallinity. Additionally, the water contact angle was determined to characterise surface wettability which was crucial in the analysis of protein absorption. X-ray photoelectron spectroscopy was used to indicate oxygen bonds amount on the surface. Finally, the impact of the crystallinity, and related properties were demonstrated on dermal fibroblasts' response. Cellular proliferation and expression of selected genes: α-SMA, collagen I, TIMP, integrin were analysed.

## Abstract 2

Low molecular weight PCLs revealed higher crystallinity, higher chain mobility at the surface, lower polarity and related higher hydrophobicity. Crystallinity and related properties decide about gene expression.

## Graphical abstract

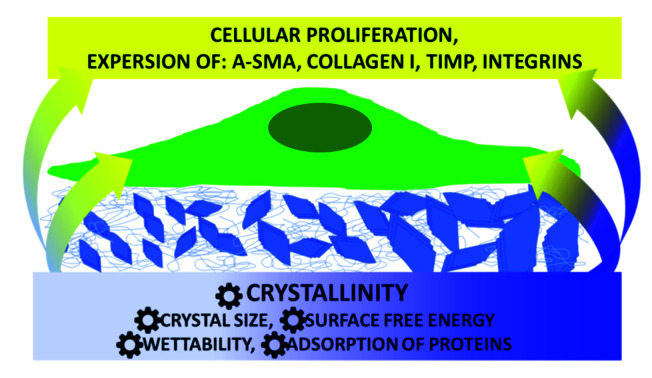

## 1. Introduction

Surface science has played an important role in biomaterials research for more than two decades. It was recognised early on that the factors governing protein adsorption and cellular adhesion must be related to the factors that mediate or control cellular response.^1^

Understanding of cell–substrate interactions is crucial for functional tailoring of scaffolds for regenerative medicine. Surface properties of polymers show an influence on tissue interface dynamics from cells seeding until final disposition.^2^ Integrins with other receptors are responsible for cell–substrate interaction. The process of identifying and understanding the role of each of the signalling pathways activated as a result of interaction between cells and substrate molecules is still incomplete.^3^*In vivo* within seconds, non-specific protein adsorption occurs on the surface. Implanted biomaterials rapidly acquire a layer of plasma proteins, *e.g.*: albumin, fibrinogen, fibronectin, and vitronectin.^4,5^ The primary cellular recognition and response of the immune systems begin when protein is adsorbed on the surface.^6^ The amount of proteins adsorbed on the surface proteins is influenced by material surface properties such as roughness, chemical structure, wettability and interactions between material and cells, such as van der Waals, polar and ionic interactions.^2,6^

As noted above, the presence of electrostatic, van der Waals, polar and hydrophobic interactions at the surface of biomaterials influences the degree and type of protein adsorption at an interface, which has a significant impact on the biological response, including the foreign body reaction. This interaction promotes cell adhesion or is a restricted barrier for cell adhesion and spreading.^6^ Enthalpic forces such as attractive Coulomb and van der Waals interactions between proteins and surfaces as well as entropic interactions regulate the standard Gibbs energy of adsorption that determines the degree of protein adsorption on the surface of biomaterials and tissue regeneration.^7^

The crystallinity degree of semicrystalline polymers affects surface properties related to proteins and cells adhesion: surface free energy, wettability, as well as types and strength of chemical bonds.^8^ Fabrication of semicrystalline polymeric substrates and scaffold take place with rapid cooling (*e.g.* fused deposition modelling, extrusion) or rapid solvent evaporation of the solvent (*e.g.* electrospinning process). Therefore, polymeric chains are far from energetically favourable orientations and thermodynamic equilibrium. Usually crystals formed in such conditions consist of chains with relative high-energy conformations. A high degree of supercooling reduces crystallinity, while the length of molecules intensifies entanglement and formation of smaller/more defected crystals.^9,10^ Finally, weak crystals decide about lower surface polarity and lower protein adsorption.^11^ The annealing process contributes to an increase of crystallinity, chain relaxation and decreases of chains' energy.^12^ Additionally, during crystallization, the chain-end segregation causes enrichment of short chains near the surface, since they have more ends per unit volume which may influence surface free energy.^13^

A review of the literature confirms the effect of crystallinity on cellular response. However, in most of the data, it is difficult to distinguish the main factor responsible for better cell proliferation or adhesion. Besides crystallinity, some other factors like topography (roughness), pore size, or chemistry were changed, interfering with effects related to crystallinity. The influence of crystallinity of poly(l-lactide) (PLLA) was analysed several times.^9,14–18^ The gradient of crystallinity of the PLLA was created by annealing, however, an unintentional increase of roughness appeared also. There is no possibility to answer the question whether cells number increased with crystallinity or roughness.^15^ Hepatocyte spreading and 3T3 fibroblasts proliferation were more efficiently on amorphous PLLA subtracts.^14^ What interesting, on crystal PLLA firms, cells losing affinity for substrate formed tight cell–cell aggregates or spheroids. In another study, amorphous poly(d,l-lactic acid) (PDLLA) was analysed under *in vitro* conditions with murine osteoblast-like cells.^16^ Analyses indicate similar adhesion on PDLLA/PLLA blends with different compositions; however, the proliferation rate was faster on the PDLLA-dominant sample (amorphous phase). Animal studies showed that tissue ingrowth is faster into amorphous PLLA scaffolds than into crystalline PLLA scaffolds.^17^ Cellular response was analysed on PLLA on form of nanotubes also.^18^ Dermal fibroblasts were found to attach readily to PLLA nanotubes but not to films of the same material. Additionally, crystalline PLLA nanotubes displayed greater cellular attachment compared to amorphous structures. In the case of PCL, the influence of and crystallinity and molecular weight on cellular response were analysed on electrospun nonwovens.^9^ Fibroblasts proliferation after *in vitro* incubation up to 7 days was highest on moderate molecular weight PCL (80 kDa) and moderate crystallinity (50.5%). It should be noted that apart from the gradient of the degree of crystallinity, the morphology and roughness of the substrate were also differentiated, so this article does not answer the question of whether crystallinity is the key factor in cellular proliferation. Besides various analysis, the origin and mechanism of distinct cell responses to crystallisation-induced surface characteristics compared with amorphous, smooth polymer substrates are still unclear. Cell adhesion and proliferation results are not always consistent, especially among different semicrystalline polymeric systems.^19^ Despite the great interest in PCL in medicine, no publications are showing the effect of the crystal phase on surface properties and cellular response. The novelty of this article is to determine the outcome of PCL crystallinity on selected properties in terms of cellular response.

The research aims to efficiently tailor the crystallinity degree of polycaprolactone and estimate its influence on cells–substrate interaction. Impact of the crystallinity on selected surface parameters: surface tension energy, chemical bonds concentration were determined. Wettability and adsorption rate of the protein were measured as a factor determining cells–substrates interaction. Finally, analysis of human dermal fibroblasts morphology, proliferation and several genes expression: α-SMA, collagen I, TIMP, integrin α-5 were verified. Expression of integrin's and collagen confirmed cell-substrate adhesion, cellular growth and ECM synthesis potential. α-SMA expression revealed the potential of using PCL in wound healing.

## 2. Materials and characterisation

### 2.1 Materials

Polycaprolactone (PCL) with an average molecular weight of Mn = 45 kDa and 80 kDa (Sigma-Aldrich Co) were used.

### 2.2 Sample preparation

PCL substrates of 10 × 10 × 0.08 cm^3^ were prepared by compressing PCL pellets in a mould *via* a hydraulic hot press at a pressure of 0.6 MPa for 5 min at 110 °C. After the compression, the PCL substrates were removed immediately from the mould and quenched to 0 °C in a container with a mixture of ice and water. Substrates formed from PCL 45 kDa and PCL 80 kDa had abbreviations PCL45, PCL80 respectively. Those substrates, after annealing in vapour during 72 h in 38 °C had the abbreviation PCL45w, PCL80w.

### 2.3 Characterization of subtracts

#### 2.3.1 Crystallinity and supermolecular structure analysis

Crystallinity was determined from DSC (Differential Scanning Calorimetry) scans. Analysis was done on the PerkinElmer Pyris 1 calorimeter with Intracooler 2P from the first heating scan. Samples (*ca.* 5 mg) were heated from 10 °C to 150 °C at a rate of 10 K min^−1^. The crystallinity of PCL was evaluated from first heating using the equation:
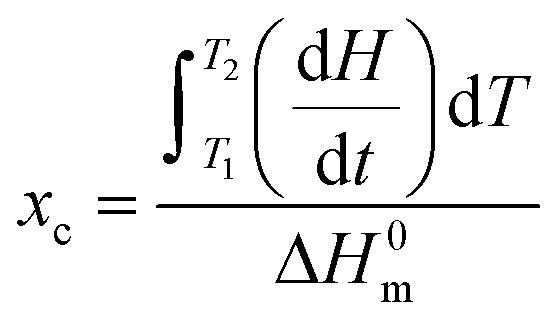
where d*H*/d*t* is the heat flow per unit mass of PCL while Δ*H*^0^_m_ is the specific heat of fusion of 100% crystalline PCL taken as 135 J g^−1^.^20^ Temperature and heat calibration were performed using indium as reference material. Melting temperature and crystallinity were taken as an average value from three measurements.

Wide angle X-ray scattering (WAXS) was applied for the analysis of crystallinity and nanostructure of the PCL fibres according to previously used methodology.^21^ WAXS measurements were performed using Bruker D8 Discover diffractometer operated at the voltage of 40 kV, current of 20 mA and CuKα radiation with a wavelength of 0.1542 nm. All measurements were performed in reflection mode at room temperature (RT). The angular range of measurements, 2*Θ*, was between 5° and 35°, with a step of 0.01° and a time of data accumulation at an angular point of 0.2 s. Considering overlapping of diffraction peaks from crystal peaks of PCL, peaks on WAXS radial profiles from crystal and amorphous phases were deconvoluted using Pearson VII and Gauss function, respectively. From this data, the degree of crystallinity, position of main peaks from crystallographic planes and crystallite sizes in directions perpendicular to (110), (111) and (200) were determined.

The crystallite size *L*_*hkl*_ perpendicular to the (*hkl*) plane was calculated using Scherrer equation:^21^
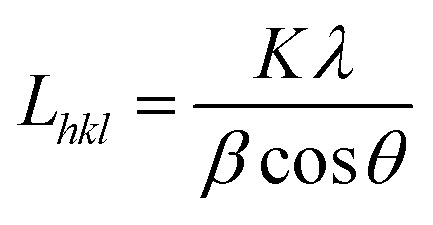
where *K* (=0.9) is the Scherrer constant, *β* is line broadening at half the maximum intensity, *λ* is the wavelength of the X-rays, and *θ* is the Bragg angle. Estimated uncertainties of crystallinity and crystal size from WAXS are 2–5%.

#### 2.3.2 Wettability and surface tension energy determination

The static contact angle of 5 μl distilled water on the subtracts' surface was measured by optical contact meter equipment (Kruss GmbH, D). Surface free energy (SFE) was determined in the Kaelble–Owens–Wendt method. The following equation allows one to determine the surface tension:^22^*γ*_s_ = *γ*^d^_s_ + *γ*^p^_s_where *γ*_s_ is the SFE *γ*^d^_S_ is the dispersion component of SFE and *γ*^p^_s_ is the polar component of SFE. To determine the SFE, two measured liquids (bipolar-water and polar-diiodomethane) whose surface tension and polar and dispersion components of SFE are known are used. Distilled water is a highly polar liquid as its polar component is 51 mJ m^−2^ with a total SFE 72.8 mJ m^−2^.^22^ The components of diiodomethane SFE are as follows: polar- 2.4 mJ m^−2^, dispersive −48.6 mJ m^−2^.^22^ Components *γ*^d^_s_ and *γ*^p^_s_ of the examined materials may be calculated from:
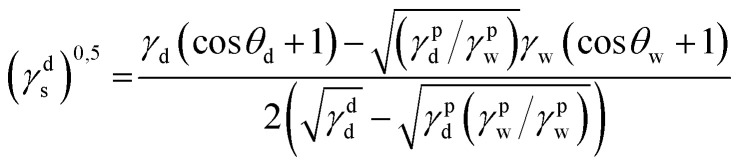

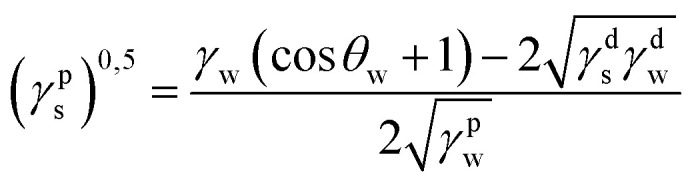
where *γ*^d^_S_ is the dispersive component of SFE of the examined materials, *γ*^p^_s_ is the polar component of SFE of the examined materials, *γ*_d_ is the free SFE of diiodomethane, *γ*^d^_d_ is the dispersive component of diiodomethane surface energy, *γ*^p^_d_ is the polar component of diiodomethane SFE, *γ*_w_ is the SFE of water, *γ*^d^_w_ is the dispersive component of water SFE, *γ*^p^_w_ is the polar component of water SFE, *Θ*_d_ is the contact angle of diiodomethane and *Θ*_w_ is the contact angle of water.

Surface polarity (*X*_p_) was defined following Wu's method^23^ as:
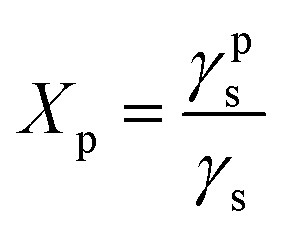


#### 2.3.3 Quantification of fibronectin adhesion

All substrates: PCL 45, PCL 45w, PCL80 and PCL80w were kept in fibronectin 40 mg L^−1^ solution for 24 h at room temperature. Aliquots of the 40 mg L^−1^ solutions of fibronectin (4, 20, 50, 100 and 200 μL) were added to a 96-well plate, and a PBS buffer was added to bring the total sample volume to 200 μL. Blank samples of 200 μL of buffer were prepared in triplicate. Fresh BCA reagent BCA assay kit (QuantiPro™, for 0.5–30 μg ml^−1^ protein conc.) was prepared following the manufacturer's instructions, and 100 μL was added to 100 μL in each well, mixed by shaking, and incubated at 37 °C for 2 h. The absorbance at 562 nm was measured immediately with a microplate spectrophotometer (Fluoroskan Ascent, Thermo Scientific). The absorbance values were corrected by subtracting the average absorbance of the blank samples. The normalised absorbance values were plotted *versus* the mass concentration (g L^−1^).

#### 2.3.4 Analysis of chemical bonds

Surface properties were determined by X-ray photoelectron spectroscopy (XPS) (PHI LS 5600) with a MgKα X-ray source. The energy resolution was set to 0.8 eV per step at pass energy of 187.85 eV for survey scans and 0.25 eV per step and 58.7 eV pass energy for region scans. The X-ray beam was operated at a current of 25 mA and an acceleration voltage of 13 kV. Charge effects were corrected using carbon C 1s = 284.5 eV. The photoelectron transitions of C 1s, and O 1s were selected for the determinations of the concentrations and chemical shifts using the region scans. The O 1s spectra have been decomposed into two distinct peaks: the first one was attributed to the oxygen singly bonded with the carbon (–O–C) at 533.58 eV and the second was the oxygen doubly bonded with the carbon (–O=C–) at 532.11 eV. Estimated uncertainties of concentration results from XPS are 5–10%. The concentrations of the surface elements and the fittings were performed using CasaXPS software.

### 2.4 Characterisation of cells–subtracts interactions

#### 2.4.1 Cells culture

Skin biopsy specimens were obtained by punch biopsies (6 mm^3^) from healthy donors undergoing corrective abdominal reduction (plastic) surgery. The study was approved by the ethics committee (ethical approval no. 13/NE/0089) and written informed consent was obtained from all patients. All methods were carried out in accordance with the approved guidelines. Dermal fibroblasts were cultured from skin biopsies as described before,^24^ and cultured in RPMI (Invitrogen) supplemented with penicillin (100 units per ml), streptomycin (100 μg ml^−1^), l-glutamine (2 mM) (all Sigma Aldrich) and 10% FBC 37 °C in an atmosphere containing 5% CO_2_. Discs cut from PCL films by cork borer were fixed into 24-well tissue culture plates. PCL substrates were sterilized with ethanol and UV (wavelength 360 nm) for 30 min. Dermal fibroblasts were seeded onto 24-well tissue culture plates or PCL substrates at the concentration of 5–6 × 10^4^ cells per ml and cultured for 24 h (day 1) or 72 h (day 3). Additionally, some fibroblasts were treated with 15 ng ml^−1^ human recombinant TGF-β (240-B-002, R&D) for 24 h or for 72 h as a positive control for matrix formation.

#### 2.4.2 Fibroblasts morphology and adhesion

For immunohistochemical staining, cells were washed once with phosphate buffered saline (PBS) without glucose, fixed in 4% paraformaldehyde plus 0.1% Triton-X for 5 min and washed again with PBS. Then actin skeleton and nucleus were stained with ActinGreen™ and NucBlue™ Reagent (Thermo Scientific), respectively. The PCL samples in comparison to TCP (Tissue Culture Plastic) were illustrated using a fluorescent microscope (Leica DMI3000B, Leica Microsystems).

#### 2.4.3 Proliferation and cytotoxicity assay

CellTiter-Glo® Luminescent Assay (Promega) for cell viability and cytotoxicity was performed directly in the cell culture in triplicate according to the manufacturer's protocol and analysed using Glomax multi detection system (Promega). This bioluminescence assay is a sensitive method of determining the number of viable cells in culture based on quantitation of the present adenosine triphosphate (ATP). ATP is an energy storage molecule, therefore can be used as an indicator of metabolically active cells. This assay measures the light that is emitted during luciferin–luciferase reaction. Cellular ATP provides energy for this reaction and the light intensity is linearly related to ATP concentration and subsequently to the cell number.

#### 2.4.4 Gene expression study

RNA from freshly isolated dermal fibroblast was obtained using the RNeasy Mini Kit (74104, Qiagen) according to the manufacturer's protocol. RNA (150–200 ng) was reverse transcribed to cDNA with the use of random primers reverse transcriptase enzyme (Life Technologies), according to the manufacturer's protocol. Specific primers and SYBR® Green JumpStart™ Taq ReadyMix™ (S4438, Sigma) or TaqMan® Gene Expression Master Mix (Thermos Fisher Scientific) probes and TaqMan™ Gene Expression Master Mix (4370048) were used according to the manufacturer's protocol. The following forward and reverse primers were used for TIMP-1 – For-5′-GAC GGC CTT CTG CAA TTC C-3′, Rev-5′-GTG GTC TGG TTG ACT TCT G-3′, 18S – For-5′-GAA TGG CTC ATT AAA TCA GTT ATG G-3′, Rev-5′-TAT TAG CTC TAG AAT TAC CAC AGT TAT CC-3′, collagen 1 a: forward 5′-CAA GAG GAA GGC CAA GTC GAG G-3′, reverse 5′-CGT TGT CGC AGA CGC AGA T-3′. TaqMan probes: integrin α-5 (ITGA5) TaqMan Gene Expression Assays (Hs00233743_m1), amplicon length 86, collagen type I α1 (COL1A1) TaqMan Gene Expression Assays (Hs00164004_m1), amplicon length 66, α-smooth muscle actin (α-SMA/ACTA2) TaqMan Gene Expression Assays (Hs00426835_g1), amplicon length 105, 18S TaqMan Gene Expression Assays (Hs99999901_s1), amplicon length 187, all form Thermo Fisher Scientific were used. Samples were analysed in triplicate using the Quant Studio 5 qRT-PCR machine (Thermo Fisher Scientific) and programme designated for Quant Studio 5. Expression levels relative to the average untreated fibroblasts (TCP) (arbitrarily set at 1) were calculated using the following equation: (2^Delta Delta CT)^−1^ all normalised to 18S housekeeping gene.

#### 2.4.5 Statistical analysis

Quantitative data of *X*_c_, *T*_m_, surface tension, contact angle, fibronectin adsorption were expressed as mean ± SD. Data were compared using a one-way ANOVA test and analysis of variance (Tukey's test). The data were considered as significantly different when *p* < 0.05. In case of *in vitro* analysis, the differences between the groups were tested for their statistical significance by non-parametric two tailed *T*-test using GraphPad Prism (GraphPad Software, San Diego, CA). A *p* value of less than 0.05 was considered statistically significant; *p* values are expressed as follows: ns for not significant; 0.05 > *p*> 0.01 as*; 0.01 > *p*> 0.001 as**; *p* < 0.001 as***. In order to confirm normal distribution D'Agostino–Pearson test has been used prior to *t*-test analysis.

## 3. Results

**Fig. 1:**
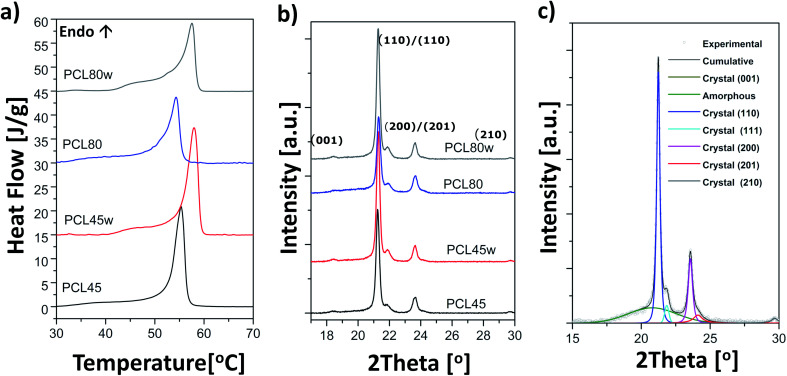
(a) Thermograms from DSC recorded as the first heating scans of PCL substrates: PCL45, PCL45w, PCL80, PCL80w; (b) acattering profiles from WAXS taken in RT of PCL substrates; (c) fitting of scattering profile of PCL45.

Polycaprolactone (PCL) substrates that differ in molecular weight and crystallinity degree (*X*_c_) were prepared. Crystallinity degree and melting temperatures determined from DSC thermograms (Fig. 1a) and crystallinity degree and crystallite sizes from WAXS scattering profiles (Fig. 1b) are shown in Table 1. Our WAXS profiles of PCL indicate diffraction from crystal structure with maximum at 2*θ* = 18.3, 21.4°, 21.9°, 23.6 and 29.7° corresponding to (001), (110), (111), (200)/(201), and (210)/(201) lattice planes (Fig. 1b and c), respectively, and the maximum of the amorphous halo at 2*θ* = 21°.^25^

**Table 1:** Characteristics of PCL45, PCL45w, PCL80, PCL80w. Crystallinity degree determined with DSC (*X*_c DSC_), melting temperature (*T*_m_), crystallinity degree determined with WAXS (*X*_c WAXS_), crystallite size in (110), (111) and (200) planes in PCL crystal lattices-L(110), L(111), L(200)

	PCL45	PCL45w	PCL80	PCL80w
*X*_c DSC_ [%]	58.0 ± 0.3	62.2 ± 0.4	49.3 ± 0.4	51.8 ± 0.3
*T*_m_ [^o^C]	55.3 ± 0.2	57.9 ± 0.2	54.3 ± 0.2	57.5 ± 0.2
*X*_c WAXS_ [%]	56.5	62.1	47.7	52.5
L(110) [nm]	33.86	35.56	31.61	32.29
L(111) [nm]	20.79	21.97	18.24	22.96
L(200) [nm]	24.55	25.61	24.54	24.58

Low molecular weight PCL (PCL45) indicated higher crystallinity than PCL with high molecular weight (PCL80). Substrate annealing contributed to the increase in *X*_c DSC_ and *X*_c WAXS_ and melting temperatures (PCL45w and PCL80w) (Fig. 1c and Table 1). Additionally, there are differences in crystallite size perpendicular to the planes (110), (111) and (200) (Table 1). These planes correspond to an orthorhombic unit cell with specific *a* = 7.48 Å, *b* = 4.98 Å, *c* = 17.26 Å sizes.^26^ Slightly higher of L(110), L(111), L(200) are observed for PCL45 than for PCL80, and for annealed samples. Those WAXS trends in crystal size correspond with relationships between melting temperatures of particular samples with higher values for PCL45 and for samples after annealing.

**Fig. 2:**
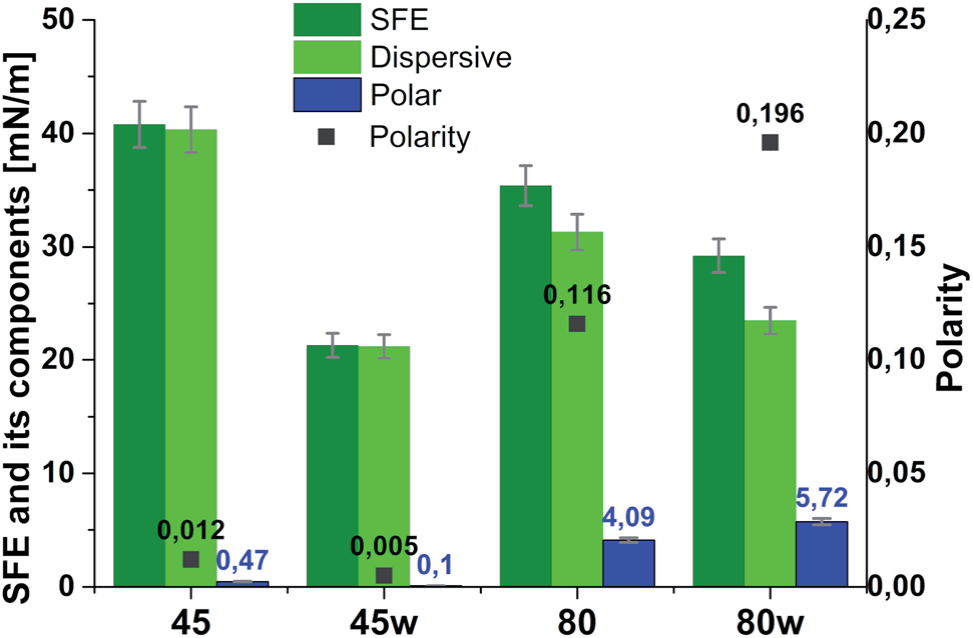
Surface free energy (SFE), its dispersive component, polar component and polarity of analysed substrates.

From the perspective of protein adsorption in biomedical applications, surface free energy (SFE) was determined. SFE is related to physical property of the polymer that can affect its wettability and therefore its adhesion to the surrounding tissues.^27^ The Kaelble–Owens–Wendt method was used to determine dispersion components and polar SFE. This calculation is related to the Bethelot hypothesis which claims that interactions between molecules of two substances, present in their surface layer, are equal to the geometric mean of intermolecular interactions within each substance.^22^ To determine the SFE, two measured liquids (bipolar-water and polar-diiodomethane) were used. In Fig. 1, SFE, dispersive and polar parts were reported. SFE of analysed substrates decreases as follows PCL45 < PCL80 < PCL80w < PCL45w. The highest SFE was observed for substrate performed from PCL with low molecular weight (42 kDa) and it dropped *c.a.* 50% after annealing (PCL45w). The PCL's SFE with high molecular weight (80 kDa) is 25% lower than PCL45 (Fig. 2).

**Fig. 3:**
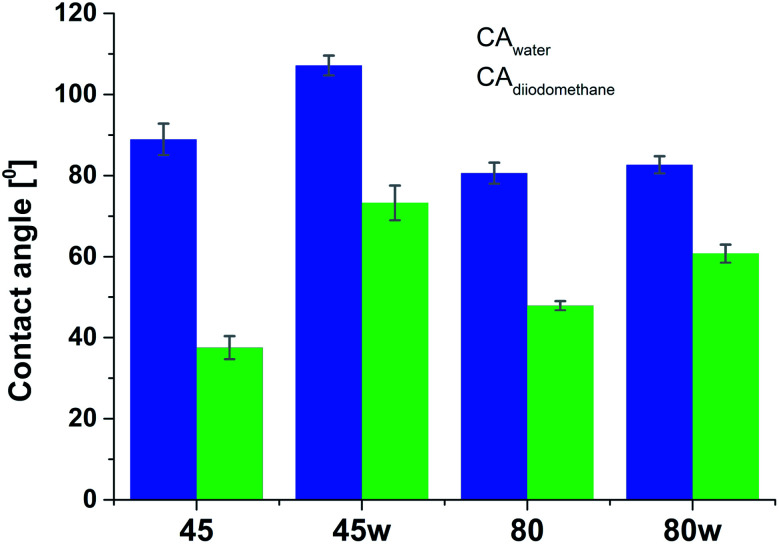
Water and diiodometane contact angles (CA_water_ and CA_diodometane_) of PCL substrates.

According to Kaelble–Owens–Wendt method, SFE is split into a dispersive component due to van der Waals and London forces and a polar component due to hydrogen bonds and dipole–dipole interactions so that the total surface energy is the sum of these two.^2,22^ SFE (*γ*_s_) and its components: dispersion component *γ*^d^_S_ and polar component *γ*^p^_s_ were determined according equations given in Methodology. The PCL substrates' dispersive components were dominant in SFE and the dispersive component decreased as follow PCL45 < PCL80 < PCL80w < PCL45w. Additionally, they follow direction changes of contact angle measured with nonpolar diiodomethane (CA_diodomethane_) (Fig. 3). The contact angle is an equilibrium between the force with which the molecules of the drop liquid are being attracted to each other (cohesive force) and the attraction of the liquid molecules for the surface (an adhesive force).^22^ PCL45 and PCL45w indicated a much lower polar contact angle than PCL80 and PCL80w, reflected in the water contact angle (CA_water_). Therefore, surface polarity (*X*_p_) was of PCL80 and PCL80w was nearly 2-fold higher than PCL45w.

**Fig. 4:**
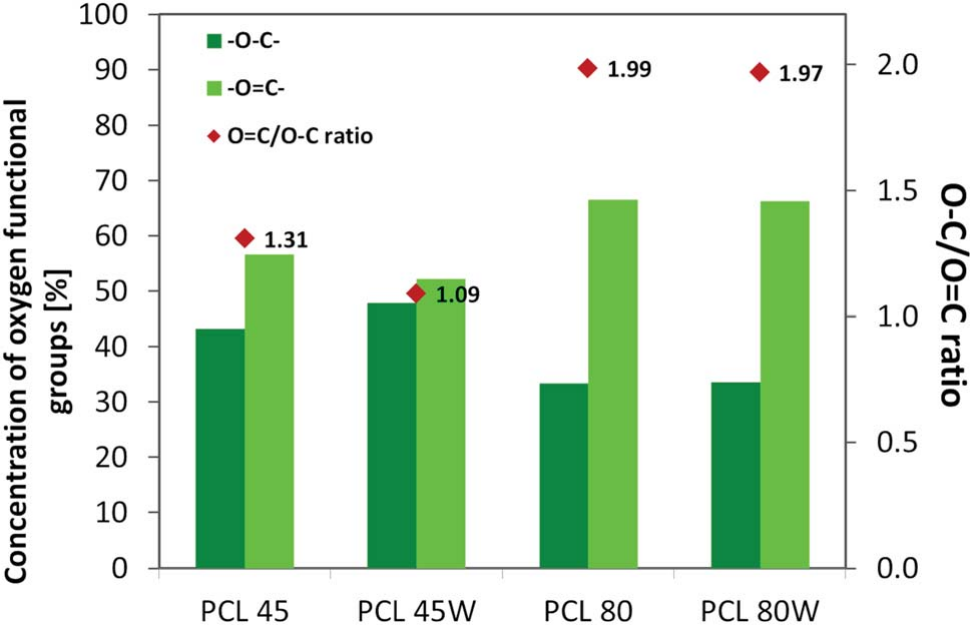
XPS results for O 1s region for analysed substrates showing relative at% of oxygen in –O=C–, –O–C– bonds and intensity ratio of –O=C–/–O–C–.

XPS is a surface-sensitive quantitative spectroscopic technique based on the photoelectric effect that can identify the elements present on a polymer surface, as well as their chemical state.^2^ The local bonding environment of an atomic species in question from the topmost few nanometres of the substrates makes XPS a unique and valuable tool for understanding the chemistry of the surface.^2^ Deconvolution of O1s spectra XPS spectra revealed a variation of selected bonding on the surface. Low molecular weight PCL45 indicated a higher –O–C concentration than PCL80. The annealing process slightly enhances the –O–C amount on the substrate surface (Fig. 4). The intensity ratio of –O=C–/–O–C– was nearly 2-fold higher for PCL 80 and PCL 80w than for PCL45w. Additionally, annealed samples indicated a higher ratio of O/C: PCL45w *vs.* PCL45-0.28 *vs.* 0.29 and PCL80w *vs.* PCL80-0.21 *vs.* 0.23.

**Fig. 5:**
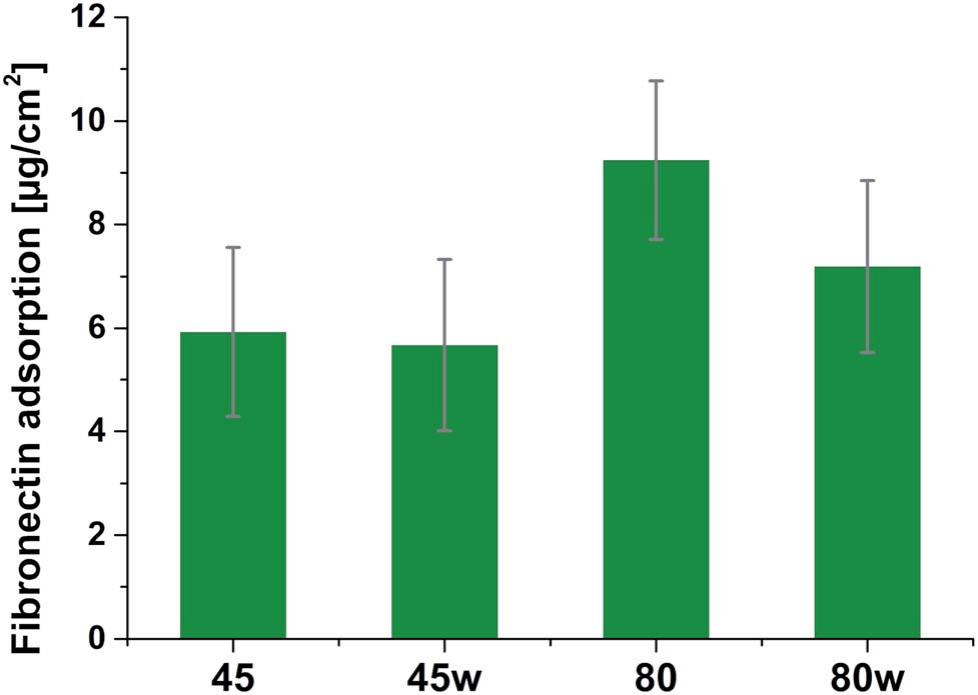
Absorption of fibronectin on the surface of PCL substrates.

Protein adsorption to surfaces involves multiple electrostatic, hydrophobic, hydrogen bonding, and van der Waals interactions.^28^ Fibronectin was chosen to analyse the adhesion ability of all PCL substrates (Fig. 5). The hydrophilic surface of the PCL80 and PCL80w with the (Fig. 9) highest ratio of O=C/O–C were preferred for fibronectin absorption. There was no statistical difference in adsorption on other substrates: PCL45, PCL45w (Fig. 4).

**Fig. 6:**
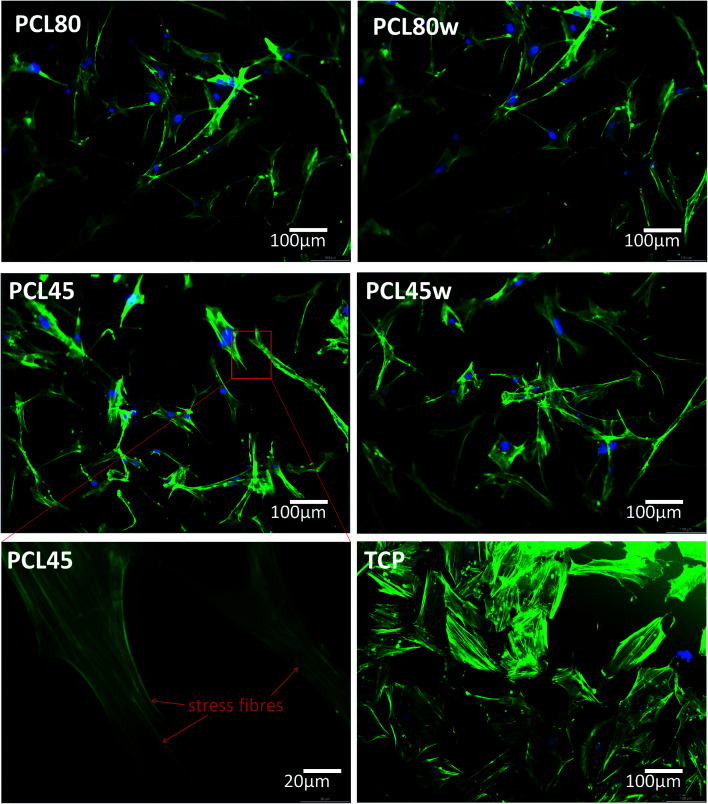
Morphology of primary fibroblasts visualised on fluorescence microscope after 3 days of cultivation on PCL45, PCL45w, PCL80 and PCL80w in comparison to TCP (Tissue Culture Plastic). As an example, stress fibres were of fibroblasts seeded on PCL45 were shown. Blue-nucleus, green-actin skeleton.

Cellular morphology consists of numerous action filaments forming filopodia and lamelopodia. Those components facilitate cell-biomaterial communication and migration.^29^Fig. 6 illustrates human dermal fibroblasts seeded on PCL subtracts as well as on TCP as a control. The actin–nuclei staining at day 3 showed that cells adhere to all substrates.

**Fig. 7:**
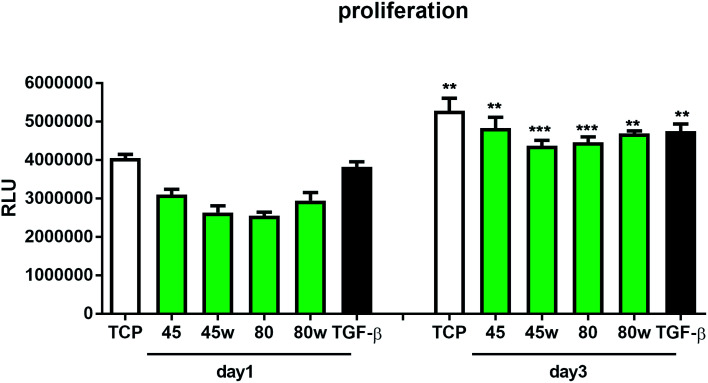
The effect of PCL substrate properties on fibroblasts proliferation. HC fibroblasts (*n* = 4) were cultivated on tissue plastics or polymers for 24 h or 72 h and ATP release from lysed cells was measured using bioluminescence assay (Relative Light Unit – RLU). Statistical analysis was performed between cells seeded on the specific polymer or plastic from day1 *vs.* day 3 and represented as * = *p* < 0.05; ** = *p* < 0.01; *** = *p* < 0.001.

To determine the proliferation degree of primary fibroblasts exposed to different polymeric substrates, CellTiter-Glo® Luminescent Assay was performed. This bioluminescence assay is a sensitive method of determining the number of metabolically active cells based on their ATP release during cell lysis. It can be seen that in the case of 72 h (day 3) following exposure to different variants of substrates, cell proliferation significantly increased compared to cells incubated for 24 h (day 1). This suggests exposure onto substrates did not affect cellular cytotoxicity and even significantly enhanced cell proliferation following 72 h incubation (Fig. 7). In this experiment, we used primary dermal fibroblasts in order to retain the original *in vivo* characteristics of the cells. Of note, primary human fibroblast treated with TGF-β also significantly increased the proliferation ratio following TGF-β activation in day 3 compared to TGF-β activation in day 1. TGF-β stimulation experiment was used as a positive control for matrix formation in the further experiments.

**Fig. 8:**
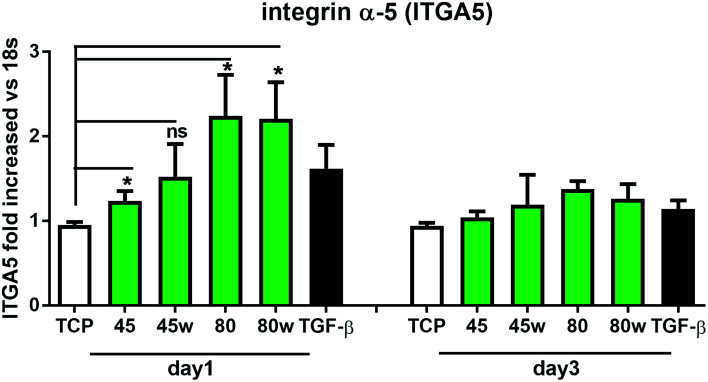
The effect of PCL substrate properties on integrin α-5 (ITGA5) expression. HC fibroblasts (*n* = 7) were cultivated on tissue plastics or polymers for 24 h or 72 h, and collagen expression was measured by quantitative polymerase chain reaction (qPCR). Statistical analysis was performed between cells seeded onto TCP (cells on plastic) *vs.* substrates 40, 45, 80 and 80w in day1 and represented as * = *p* < 0.05; ns = not significant.

Integrins with other receptors are responsible for cell–substrate interaction. The process of identifying and understanding the role of each of the signalling pathways activated as a result of interaction between cells and natural ECM molecules is still incomplete.^30^ Expression of integrin (ITGA5), which is a key mediator in the cell adhesion and in the proper formation of tissue architecture^31^ was analysed. In Fig. 8 it can be seen that the expression of ITGA5 was significantly increased in fibroblasts seeded on PCL45, 80 and 80w substrates in day 1. At day 3, ITGA5 expression on PCLs was similar to cells seeded on TCP. Although it did not reach statistical significance probably due to the limited number of substrates, a similar tendency of increased ITGA5 expression in day 1 was also observed on PCL45w. This suggests that ITGA5 is a crucial adhesion molecule during the initial stages of cell attachment to polymers and diminishes when matrix architecture begins to form.

**Fig. 9:**
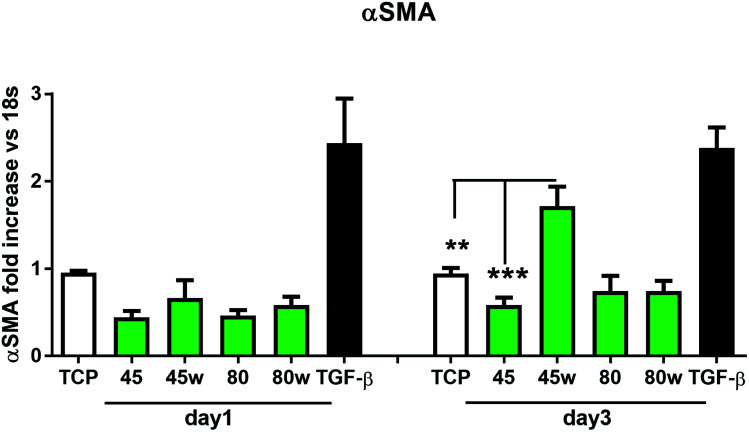
The effect of PCL substrate properties on α-SMA expression. HC fibroblasts (*n* = 9) were cultivated on tissue plastics or polymers for 24 h or 72 h and α-SMA was measured by quantitative polymerase chain reaction (qPCR). Statistical analysis was performed between cells seeded onto PCL45w *vs.* PCL45 and TCP (cells on plastic) in day 3 and represented as * = *p* < 0.05; **= *p* < 0.01; *** = *p* < 0.001.

**Fig. 10:**
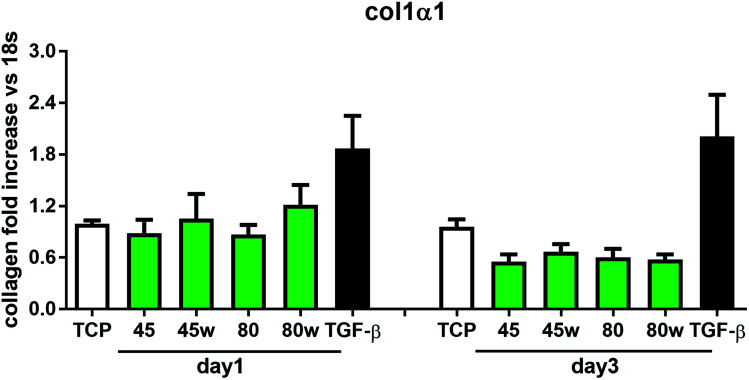
The effect of PCL substrate properties on collagen type I-α 1 expression. HC fibroblasts (*n* = 9) were cultivated on tissue plastics or PCLs for 24 h or 72 h and collagen expression was measured by quantitative polymerase chain reaction (qPCR).

**Fig. 11:**
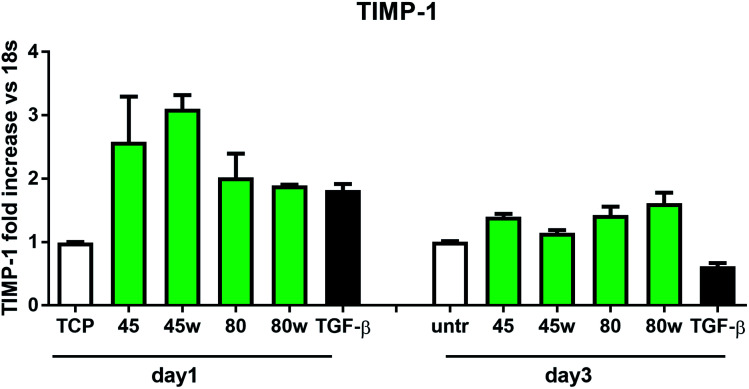
The effect of PCL substrate properties on TIMP-1 expression. HC fibroblasts (*n* = 2) were cultivated on tissue plastics or PCLs for 24 h or 72 h and TIMP-1 expression was measured by quantitative polymerase chain reaction (qPCR).

In healthy tissues, collagen fibres forming the ECM are produced from procollagen synthesised by fibroblasts. In pathological conditions, uncontrolled collagen degradation occurs under the impact of a series of proteolytic enzymes, metalloproteinases (MMPs), including collagenase MMP1, MMP8 and MMP13. They initiate the process of degradation by alpha decay chains of collagen I and III. Subsequently, gelatinase MMP2 and MMP9 degrade the resulting fragments.^32^ The activity of MMPs is regulated by their tissue inhibitors Tissue Inhibitors of Metallproteinases, (TIMPs). However, overexpression of MMPs results in imbalance between TIMPs/MMPs and may be involved in disease progression. On the other hand, collagen degradation products, in turn, stimulate fibroblasts to form extracellular matrix proteins. Where collagen fibres formation dominates its degradation, prepared positive feedback leading to fibrosis. Alpha-smooth muscle actin (α-SMA) is a marker of myofibroblast formation. It is highly conserved protein that is involved in cell motility, structure and integrity.^33^ Therefore, we also measured the gene expression of matrix proteins including α-SMA (Fig. 9), collagen (Fig. 10), TIMP-1 (Fig. 11) in primary dermal fibroblasts. As a positive control for ECM-induction we used TGF-β. It is well known that TGF-β mediates in ECM production and maintenance of tissue homeostasis.^24,34,35^ It can be seen that α-SMA expression was significantly increased only in fibroblasts cultivated on PCL45w scaffold compared to PCL45 (2.9-fold, *p* = 0.0002) or TCP (1.8-fold *p* = 0.0044) in day 3 (Fig. 9). Collagen expression was slightly higher on annealed samples at day 1 and on PCL45w at day 3 (Fig. 10). Surprisingly, no difference was found in the expression level of collagen upon incubation on all types of PCLs in day 1 and even slightly drop in day 3 (Fig. 10). At the same time, TIMP-1 expression was the highest on PCL45w at day 1 (Fig. 11). This suggests that polymer PCL45w provides the best conditions for TIMP-1 expression in day 1 and α-SMA expression in day 3, which may be used as an early indicator of the ECM development.

## 4. Discussion

In this study, we tailored substrates crystallinity by differentiation of the molecular weight of the PCL and annealing. The analysis carried out by the DSC and WAXS methods showed that the degree of crystallinity of low molecular weight PCL (PCL45) is higher than PCL80. Kinetics of crystallisation of PCL was slower for higher molecular weight samples which was explained with the retarded segmental mobility of the longer chains.^9,36^

### 4.1 Crystallinity and size of crystals

PCL exhibits an orthorhombic crystal structure and nearly 2.5-fold higher lattice dimension “*c*” than lattice dimension “*a*”; therefore, it is a so-called crystal fixed polymer.^37^ No intracrystalline mobility of PCL is related to the strongly polar carbonyl groups undergoing intermolecular coupling.^32^ Similar trend was observed for high molecular weight semicrystalline PLLA, which exhibited limited crystallisation due to high density of chains entanglement presented lower chain mobility.^38^ Authors suggested that, the lower the molecular weight was, the higher the crystallisation rate of PLLA. Under an annealing process, chain mobility increased with temperature resulting in an increase of crystallinity (PCL45w, PCL80w).

The difference between crystallinity determined from DSC thermograms and WAXS scattering was related to methodology and was reported by others.^21^,^39,40^ It is evident from DSC that small/imperfect crystals were melted at low temperatures. At the same time chains, mobility increased, and recrystallization took place. Finally, the DSC melting peak results not only from original crystals but also from crystals formed dynamically during heating. It can be concluded that the crystallinity obtained from both methods was relatively close to each other.

Crystal size in planes perpendicular to (110), (110), (200) of the PCL45 were slightly lower than crystals of PCL80. Low molecular weight polymers indicate the lower density of entanglement of polymer chains, therefore larger and less defective crystals are formed.^9^ The analyzed crystal size increased with annealing in comparison to no annealed substrates. It can be indirectly related to the lamellar thickness, which explains the larger size for the annealed samples. It is expected that after annealing, the more significant change can be detected in the lamellar thickness. Annealing leads to crystal thickening and the formation of more thermodynamically stable crystals than the original crystals.^39,41^

According to the Thomson–Gibbs equation, the melting temperature (*T*_m_) of a crystallite is related to its lamellar thickness.^10^ Annealed substrates indicated higher *T*_m_ in comparison to PCL45 and PCL80, because their lamellar thickness is more increased.^39^*T*_m_ of low molecular weight is higher than *T*_m_ of high molecular weight PCL due to thicker lamellas also.^39^ An increase of crystal sizes determined using WAXS reflected a rise in lamellar thickness.

### 4.2 Properties of the surface

Our investigations revealed substrates with high molecular weight indicated lower surface energy related to lower crystallinity what is in agreement with the literature.^7,42^ Additionally, the SFE of the PCL45 was 1.5-fold higher than the surface tension of the PCL45w. One hypothesis is the gradient of spherulites size, crystallinity and crystal perfection.^43^ The skin layer is formed in a hydraulic hot press and solidified under rapid cooling in water-ice and temperature gradient. Crystallised polymer on the surface acts as insulation barrier due to its low thermal conductivity, leading to a slightly slower cooling rate in the core. Consequently, the substrate surface shows generally lower crystallinity, showing a spherulite size gradient with dimensions decreasing towards the surface.^43^

PCL substrates were formed with rapid cooling, above their *T*_g_ and their chains were not allowed to find energetically favourable orientations.^12,43^ They were far from thermodynamic equilibrium, where in this instance polymer chains have been “frozen” into high-energy conformations. The annealing process contributed to chain relaxation and decrease of chains' energy.^12^ That's why, the SFE of the PCL45 and PCL80 was 50% and 30% higher respectively, than the SFE of substrates after annealing (PCL45w and PCL80w). During annealing, chain mobility enhances total crystallinity, and fractions not accommodated in the crystalline structure might be rejected to the surface, and relaxation processes take place. Samples with low molecular weight (PCL45) indicated a higher degree of chains mobility, and the increase of SFE after annealing decreased more significantly than the SFE of the PCL80. The direction of changes in the water contact angle was identical to changes in SFE.

Additionally, XPS data confirmed a slightly higher amount of –O–C groups on annealed samples which were more hydrophilic/less hydrophobic than their no annealed equivalents (PCL45w *vs.* PCL45; PCL80w *vs.* PCL80). The chain-end segregation causes an enrichment of short chains near the surface, since they have more ends per unit volume what may influence on surface tension.^13^

### 4.3 Fibronectin adsorption

Fibronectin adsorption on the surface exhibited very different adsorption kinetics on hydrophobic and hydrophilic surfaces.^29,31,44^ We observed much higher fibronectin adsorption on the most hydrophilic surface PCL80 with a water contact angle 82°. Some reports confirm that fibronectin adsorption is partially reversible on the hydrophilic surface, but much less so on the hydrophobic surface.^42^ The polarity for high molecular weight PCL is much higher than for low molecular weight PCL, indicating strong polar contributions to surface interaction.^45^ Therefore, fibronectin adsorption was higher on PCL80 and PCL80w than on low molecular weight PCLs: PCL45 and PCL45w. Dispersive interactions were dominant in low molecular weight PCLs. Those interactions were caused by temporary fluctuations of the charge distribution in the atoms/molecules. Polar interactions actions are more stable than dispersive-hydrogen bonding *vs.* van der Waals bonding.^45^

Additionally, fibronectin adsorption was related to –C=O–/–C–O– ratio on the PCL surface. We observed the lowest intensities ratio of –C=O–/–C–O– for PCL45w with low molecular weight and highest crystallinity degree probably related to energetically favorable orientations and related thermodynamic equilibrium. The theoretical atomic ratio of –O=C–/–O–C– is 1 : 1;^46^ therefore, the surface of the PCL45w was in an equilibrium state (lowest SFE).

Fibronectin adsorption from PBS solution and cell culture in DMEM took place in pH about 7.5, so the surface of the PCL was negatively charged (isoelectric point of the PCL is *c.a.* 3.5).^47,48^ The surface of PCL80 and PCL80w were more electronegative due to the intensity ratio of –C=O–/–C–O– nearly 2-fold higher than PCL45w. Therefore fibronectin adsorption was higher on PCL80 and PCL80w. Human plasma fibronectin contains several heparin binding sites, with a high, positive charge density.^49^ Thus, fibronectin interacts with negatively-charged surfaces of PCLs through binding sites characteristic for heparin. Additionally, fibronectin might interact with the hydrophobic surface involving some of the apolar residues in the protein interior, suggesting a partial denaturation.^49^

### 4.4 Cells-subtracts interactions

The *in vitro* analysis confirmed the non-cytotoxic character of substrates in contact with human dermal fibroblasts. Interestingly, cellular proliferation was slightly higher on PCL45 and PCL80w. The reason might be more intensive protein-substrate interaction on the substrate with a higher polar component of the SFE provoking higher hydrogen bonds and dipole–dipole interactions in the early stage and therefore easier cell adhesion. Proliferation did not depend significantly on the molecular weight of the substrate which is in agreement with investigation performed on printed PCL,^50^ and on PEG with low RGD nanospacing.^51^ Proliferation degree did not follow crystallinity degrees of PCL's substrates what is in agreement with mouse fibroblasts cultivated on PLLA up to 45 h.^14^ However our investigation is in opposition to research on PLLA substrates, where cell proliferation was increased in the high molecular weight amorphous samples.^52^

After 1 day of cultivation, the expression of integrin α-5 (ITGA1)was similarly high on hydrophilic PCL80 and PCL80w. PCL 45, indicating lower crystallinity and contact angle revealed a lower integrin α-5 expression than PCL45w. Integrins are the main cell surface receptors that mediate cell–matrix adhesion.^44^ We observed higher fibronectin adsorption on substrates with wettability *c.a.* 80 degrees and high-intensity ratio of –O=C–/–O–C– (PCL80 and PCL80w). Fibronectin is an extracellular matrix (ECM) component that, through binding integrin receptors of the cell surface, acts as a critical player of the communication between the intra and the extracellular environment, thus controlling cell behavior.^44^

Collagen type I-α1 (Col1A1) expression was more efficient on annealed substrates, indicating higher crystallinity and lower surface tension (PCL45w, PCL80w). What is surprising and essential from the perspective of substrate/scaffold fabrication, molecular mass deciding about mechanical properties^53^ did not dominate collagen synthesis. Cells seeded on substrates with stiffness from 0.3–100 kPa did not reveal a significant difference in Col1A1 expression, besides drop of MMP-a expression and an increase of procollagen.^54^ Authors demonstrated a dominant role for normal tissue compliance, acting in part through autocrine PGE2, in maintaining fibroblast quiescence and revealing a feedback relationship between matrix stiffening, COX-2 suppression, and fibroblast activation that promotes and amplifies progressive fibrosis. According cellular studies made of micropatterned substrates covered with poly(dimethylsiloxane), elongated cells expressed higher collagen type I than did less stretched cells even though cell spreading area was the same.^55^ Authors suggests, normal tendon fibroblasts *in vivo* have an “optimal shape” for maximizing synthesis of type I collagen. Under *in vivo*, more intensive synthesis of collagen was observed on low molecular chitosan than on high molecular weight chitosan what was associated with a prior reduction of the inflammatory response and improved wound healing.^56^ TIMP-1 as a collagen degradation blocking factor was the most active after 1 day on samples with lower molecular weight (PCL45). An additional activity increase was observed on samples with higher crystallinity and lower wettability (PCL45w, PCL80w).

α-SMA is a marker of myofibroblasts formation which are crucial in closing the wound tissue through their capacity to produce a strong contractile force.^57^*De novo* expressed α-SMA is incorporated in stress fibers and this actin isoform plays an essential role in granulation tissue contraction.^57^ α-SMA expression was observed on samples indicating higher crystallinity and lower surface tension. The rapid increase of myoblast formation was observed after 3 days of cultivation on PCL45w indicating the highest crystallinity. Expression of α-SMA was observed on aligned fibres which revealed aligned topography and good mechanical properties.^58^ We previously proved, aligned fibres indicate higher crystallinity and molecular order than nonaligned (random) fibres.^59^ α-SMA expression on PCL45w was also in agreement with the observation of Hinz *et al.*^57^ An increase of cells expressing α-SMA was observed on silicone substrates indicating high stiffness.

## 5. Conclusions

Our results clearly indicate that the molecular weight of the polymer and related properties are essential for the expression of the integrins. Low molecular weight PCLs revealed higher crystallinity, higher chain mobility at the surface, lower polarity and related higher hydrophobicity. On those substrates, adsorption of proteins took place mainly due to van der Waals binding, which determined cellular adhesion and growth. Contrary, high molecular weight PCL revealed slightly lower crystallinity and wettability. Surface polarity contributed to hydrogen bonding with adsorbed proteins *in vitro*. Therefore, primary protein adsorption was higher and more stable than on low molecular width PCLs. High molecular weight PCLs revealed higher integrins expression and related cellular adhesion. Collagen and myofibroblast markers as ECM indicators were unaffected by molecular weight, but they were influenced by a high degree of crystallinity within a substrate formed from a polymer of the same molecular weight. At day 3, PCL45w exhibiting the highest crystallinity and lowest surface free energy indicated the highest marker of myofibroblast formation.

Fundamental understanding of cells-substrate is crucial in the fabrication of biocompatible, functional implants. However, our study confirmed that by changing crystallinity, we also change much more properties, which are crucial for cellular adhesion, proliferation and genes expression. A variety of processes influences adhesion and integrin expression in the first days of *in vitro*. Some other determined ECM synthesis and related genes expression at day 3. Therefore, during the development of implants, there is a need to control the parameters of the implant formation process, maintaining a balance between the degree of crystallinity of the polymers and the resulting properties such as surface tension, wettability or mechanical properties.

## Ethical statement

Human fibroblasts used for experiments were obtained by skin biopsy. The study was approved by the Health Research Authority (National Research Ethics Service – Sunderland, ethical approval no. 13/NE/0089), and written informed consent was obtained from all patients.

## Conflicts of interest

There are no conflicts to declare.

## Supplementary Material
